# Resin Infiltration in the Conservative Management of Incipient Dental Caries: An Evidence-Based Review

**DOI:** 10.3390/jcm15166485

**Published:** 2026-08-21

**Authors:** Gildo C. Santos, Maria Jacinta M. C. Santos

**Affiliations:** Division of Restorative Dentistry, Schulich School of Medicine and Dentistry, Western University, London, ON N6A 5C1, Canada

**Keywords:** caries risk assessment, dental caries, microinvasive dentistry, minimally invasive dentistry, permanent teeth, proximal caries, resin infiltration

## Abstract

Dental caries is a biofilm-mediated, non-communicable, multifactorial disease in which the balance between demineralization and remineralization determines whether an initial enamel lesion remains stable, arrests, or progresses toward cavitation. Contemporary caries management prioritizes preservation of tooth structure and emphasizes individualized decisions based on lesion activity, cavitation status, cleansability, radiographic depth, and patient’s caries risk. Resin infiltration has emerged as a microinvasive approach positioned between noninvasive preventive strategies and conventional restorative treatment. This evidence-based narrative review summarizes the current literature on resin infiltration for incipient, non-cavitated proximal carious lesions in permanent teeth, with emphasis on diagnosis, case selection, clinical protocol, and current evidence. A structured search of three databases (PubMed, Embase, and the Cochrane Central Register of Controlled Trials) identified randomized clinical trials, observational clinical studies, systematic reviews, narrative reviews, and relevant in vitro investigations. The strongest clinical evidence supports resin infiltration for non-cavitated proximal lesions in permanent teeth, particularly enamel and selected outer dentin lesions, where long-term studies demonstrate reduced radiographic progression. In vitro studies indicate that lesion penetration, enamel hardness, and surface roughness depend on lesion characteristics, pretreatment, and technique, and help explain the caries-arresting effect. Current evidence shows that resin infiltration should be considered a selective microinvasive option for incipient proximal caries, guided by lesion assessment and patient’s caries risk rather than a universal substitute for prevention or restoration.

## 1. Introduction

Dental caries remains one of the most prevalent chronic diseases worldwide and is now understood as a dynamic process influenced by biofilm activity, dietary carbohydrate exposure, saliva, fluoride availability, host factors, and behavioral determinants [1]. The earliest clinically detectable stages involve subsurface mineral loss before cavitation. At this stage, the surface layer may remain relatively intact while the underlying enamel becomes porous, producing the characteristic appearance of an initial lesion or white spot lesion [2]. Because the process is potentially reversible or can be arrested, early diagnosis and appropriate intervention are central to contemporary caries management, which emphasizes minimal intervention while providing individualized, tailored treatment.

The management philosophy shift in operative dentistry toward minimally invasive dentistry aims to preserve sound tissue, control disease activity, and delay or avoid entry into the restorative cycle [3]. A thorough caries risk assessment should precede treatment planning, as it allows clinicians to tailor interventions to each patient’s individual risk level. Early identification of caries risk enables timely intervention before disease progression, reducing the need for invasive treatment and helping preserve tooth structure [4,5]. Patients classified as high caries risk face a greater likelihood of developing new carious lesions and typically require more frequent preventive counseling and fluoride application, along with a higher probability of eventually needing restorative treatment [6]. Early preventive measures may avoid or postpone restorative intervention, which should be reserved for lesions that are cavitated, plaque-retentive, or no longer cleanable. Operative treatment of lesions that could otherwise be arrested or stabilized risks removing sound tooth structure unnecessarily and increases the lifetime burden of restoration replacement. For this reason, modern treatment thresholds emphasize integrating lesion activity, cavitation, cleanability, depth, patient risk, and feasibility of disease control [7].

Initial caries lesions are often classified using clinical and radiographic criteria. Although bitewing radiography is essential for approximal lesion detection, it cannot reliably determine activity or surface integrity and may underestimate demineralization extent [8,9]. In a cross-sectional study, Urzúa et al. [10] found that cavitation was uncommon in enamel lesions (E1 and E2) and increased with radiographic depth into dentin: most D1 and half of D2 lesions remained non-cavitated, whereas D3 lesions were predominantly cavitated. These findings support restorative treatment, mainly for D3 lesions, and the use of temporary tooth separation to guide management of D1 and D2 lesions. Clinical examination, temporary tooth separation, and careful assessment of lesion accessibility may therefore influence whether a lesion is managed preventively, treated micro-invasively, or restored. Given that such a large proportion of dentin-extending lesions remain non-cavitated, a treatment option capable of arresting progression without sacrificing tooth structure is needed to bridge the gap between preventive monitoring and restorative intervention. Resin infiltration was introduced to address this clinical space between prevention and restoration and has been shown to exert a protective effect against proximal lesion progression across varying follow-up periods [11,12,13,14]. This technique uses a low-viscosity, light-cured resin to penetrate porous enamel after conditioning, replacing air and water within the lesion body and forming a diffusion barrier. This barrier reduces pathways for acids and dissolved minerals, helping arrest lesion progression. For proximal lesions, where access is limited and restorative treatment would sacrifice sound tooth structure, this microinvasive diffusion barrier offers a conservative means of stabilizing the lesion.

Despite the growing body of evidence on resin infiltration, no recent evidence-based narrative review has integrated lesion selection criteria, biological rationale, clinical protocols, current clinical trial evidence, and relevant in vitro findings into a unified clinical decision-making framework focused exclusively on the management of non-cavitated proximal lesions in permanent teeth. This review addresses that gap. It differs from prior publications in that it explicitly addresses the distinction between evidence derived from permanent-tooth studies and pooled estimates that include primary dentition data, a nuance not consistently addressed in prior narrative reviews. This manuscript presents an evidence-based narrative review of resin infiltration for the management of proximal non-cavitated caries lesions in permanent teeth. It addresses diagnostic and caries-risk-based decision-making, the evidence supporting lesion arrest, the laboratory findings regarding the technique and enamel penetration, and the limitations relevant to clinical application.

## 2. Materials and Methods

This narrative review aimed to summarize evidence from randomized clinical trials, observational clinical studies, systematic reviews, meta-analyses, and relevant in vitro studies. This review follows a narrative rather than systematic methodology; the search and selection process are reported transparently to support reproducibility and credibility of the evidence synthesis.

Eligible articles were retrieved from PubMed (MEDLINE), Embase, and the Cochrane Central Register of Controlled Trials (CENTRAL) for the period January 2015 to December 2026. No language restriction was applied where an English-language abstract provided sufficient extractable data. Two search strategies were used, a main clinical and outcome search, and a supplementary search targeting in vitro mechanical and material outcomes. The main clinical and outcome search employed the following string:

(“Dental Caries”[Mesh] OR caries[tiab] OR “initial caries”[tiab] OR “incipient caries”[tiab] OR “non-cavitated lesion”[tiab] OR “early enamel lesion”[tiab] OR “white spot lesion”[tiab] OR ICDAS[tiab]) AND (“resin infiltration”[tiab] OR “caries infiltration”[tiab] OR “resin infiltrant”[tiab] OR “infiltrative resin”[tiab] OR ICON[tiab])

A supplementary search targeting in vitro mechanical and material outcomes employed the following string:

(“resin infiltration”[tiab] OR “resin infiltrant”[tiab] OR ICON[tiab]) AND (“microhardness”[tiab] OR “surface roughness”[tiab] OR “penetration depth”[tiab] OR “enamel permeability”[tiab] OR “lesion depth”[tiab]) AND (“dental caries”[MeSH Terms] OR “enamel”[tiab])

Additional sources included citation tracking of key systematic reviews and reference list hand-searching. In total, 475 records were identified across all sources, 35 duplicates were removed, and 440 records were screened at title and abstract level. Of these, 195 full texts were assessed for eligibility, and 119 studies met the eligibility criteria and informed the evidence synthesis. Formal risk of bias scoring was not applied, consistent with the narrative review design.

Studies were included if they evaluated resin infiltration in permanent teeth and reported clinical caries outcomes (caries arrest or radiographic lesion progression at proximal surfaces) or laboratory outcomes (penetration depth, surface microhardness, surface roughness). Both in vivo and in vitro evidence were included and are reported in parallel, with in vivo evidence informing clinical effectiveness for proximal lesions and in vitro evidence informing mechanism and material behavior.

Studies were excluded if they involved only primary dentition, evaluated esthetic indications rather than caries arrest, or consisted of materials development reports without a resin infiltration comparator or relevant laboratory outcomes. Letters, editorials, conference abstracts, and case reports lacking extractable data were also excluded. Systematic reviews including both primary and permanent dentition were retained, with data extracted only for the permanent teeth subgroup.

## 3. Resin Infiltration Evolution

Resin infiltration was first investigated in the 1970s, when studies showed that adhesives could penetrate demineralized enamel and arrest lesion progression [15,16]. These studies established surface conditioning as a key component of infiltration protocols. Later work with resorcinol formaldehyde resin confirmed the concept of pore occlusion but was abandoned because of cytotoxicity concerns [16]. Subsequent studies identified the penetration coefficient as a key determinant of infiltrant performance. In artificial bovine enamel lesions, higher penetration coefficients were associated with deeper resin penetration [17]. Further research demonstrated that both penetration coefficient and resin composition influenced the ability of infiltrants to reduce lesion progression under demineralizing conditions [18]. These findings were later confirmed in natural caries lesions, where high penetration coefficient resins achieved near complete lesion infiltration, with a solvent free, TEGDMA rich formulation emerging as the preferred material [19].

The resin infiltration concept was subsequently commercialized in Germany as a dedicated microinvasive treatment for non-cavitated smooth surface and proximal lesions. Introduced in 2009, Icon (DMG) remains the only commercially available resin infiltrant identified in recent reviews. Its formulation consists primarily of the low viscosity monomer triethylene glycol dimethacrylate (TEGDMA), with ethanol as a solvent and camphorquinone as a photoinitiator, enabling deep capillary penetration into porous enamel [20]. The clinical protocol combines hydrochloric acid etching to remove the surface barrier, ethanol drying to enhance lesion desiccation, and resin application followed by light curing, creating a polymerized barrier within the lesion body [21].

High TEGDMA formulations achieved effective lesion penetration because of their low viscosity, low contact angle, and high penetration coefficient; however, their hydrophilic nature was associated with reduced mechanical properties, greater polymerization shrinkage, increased water sorption, and material degradation [20,22,23]. To address these limitations, researchers investigated alternative monomers, solvents, and additives, including BisEMA, UDMA, and acidic monomers such as PAM. Experimental studies showed that TEGDMA PAM formulations could achieve penetration comparable to Icon under some conditions without requiring separate acid etching [21,24], whereas values for TEGDMA/UDMA based blends were significantly higher than Icon [25]. Subsequent developments also targeted optical and mechanical performance. Modifying co monomer composition improved refractive index matching for lesion masking, while some experimental formulations, particularly TEGDMA/HEMA/Bis GMA blends, demonstrated higher degrees of conversion and greater flexural strength than Icon [26,27].

A key limitation of current resin infiltrants is their lack of radiopacity, making it difficult to identify previously treated lesions radiographically and document preventive intervention [21]. To address this limitation, researchers have investigated radiopaque monomers and fillers. Among these, Pedreira et al. incorporated 40% ytterbium oxide into experimental and commercial (Icon) infiltrants, achieving radiopacity equal to or greater than enamel while improving polymerization and maintaining low solubility. Although filler addition increased viscosity, the study could not determine its effect on penetration depth using microtomography and microradiography methods [28]. Similarly, Hasan et al. developed a tribromoethyl methacrylate-based infiltrant capable of full-depth penetration into artificial enamel lesions without prior HCl etching, achieving a radiopacity that matched or exceeded normal enamel and made the material clearly detectable on dental radiographs [29]. However, enhancing radiopacity may compromise infiltration performance. Some filler-containing formulations showed increased viscosity and insufficient radiopacity, while yttrium trifluoride particles reduced lesion penetration because of their large particle size [21]. Research on experimental radiopaque infiltrants remains predominantly in vitro; clinical validation in patients and long-term outcome data are still needed.

Current research has increasingly focused on incorporating antibacterial functionality into resin infiltrants to suppress S. mutans biofilms while preserving penetration capacity and material performance. Various approaches have been investigated, including quaternary ammonium monomers, ionic liquid systems, graphene oxide, and bioactive fillers. The incorporation of the pH responsive monomer DMAEM into a BisGMA/TEGDMA infiltrant significantly reduced bacterial activity and biofilm formation at concentrations up to 5% without compromising cytocompatibility or mechanical properties [30]. Similarly, the addition of graphene oxide reduced S. mutans colony counts and biofilm metabolic activity while maintaining effective infiltration of demineralized enamel [31]. An alternative strategy using a bioderived isosorbide monomer combined with a zwitterionic compound produced an infiltrant that resisted bacterial adhesion and retained this effect after simulated aging, outperforming the commercial control [32]. Another approach involved incorporating microencapsulated antibacterial ionic liquids into the resin matrix, which increased surface hydrophobicity without adversely affecting mechanical properties or biocompatibility [33]. Collectively, these findings demonstrate that antibacterial activity can be integrated into resin infiltrants through multiple chemical strategies while preserving their essential clinical properties.

Additionally, recent efforts have focused on enhancing resin infiltrants with bioactive properties that promote ion release and remineralization in addition to lesion sealing. Early studies achieved this by incorporating bioactive fillers such as Biosilicate and nanohydroxyapatite. For example, the addition of Biosilicate (5% or 10%) or nanohydroxyapatite (10%) induced calcium and phosphate deposition without impairing infiltration into white spot lesions; however, these modifications reduced degree of conversion and flexural strength while increasing solubility, particularly in Biosilicate-containing formulations [34]. More recently, a bioactive resin infiltrant containing a novel high phosphorus bioactive glass (PSC) was shown to release calcium and phosphorus ions and promote hydroxyapatite formation while maintaining physicomechanical properties, cytocompatibility, and penetration depth comparable to the commercial Icon infiltrant [35]. After 28 days in simulated body fluid, PSC treated enamel also demonstrated significantly greater cross-sectional microhardness than Icon treated enamel, suggesting that this approach may provide remineralizing benefits without the mechanical compromises observed with earlier bioactive fillers [35].

## 4. Clinical Decision-Making: Lesion Detection, Risk Assessment, and Intervention Thresholds

### 4.1. Lesion Assessment

Initial lesions are generally non-cavitated and may present as white or brown areas depending on mineral loss, surface characteristics, and extrinsic pigmentation [8,36]. Inactive lesions tend to be shiny and smooth, whereas active lesions are more often matte, chalky, and rough. Cavitation is a decisive factor because a disrupted surface can retain biofilm and may no longer be adequately controlled by topical preventive therapy alone [7]. For proximal lesions, clinical access is limited, making bitewing radiography a practical tool for detection and depth estimation. However, radiographs cannot determine lesion activity or surface integrity with certainty [9].

Based on clinical consensus and observational evidence, temporary tooth separation has been proposed as an adjunct when clinical confirmation of cavitation is needed, particularly for proximal lesions in which the radiographic image alone is uncertain [37]. Studies comparing radiographic lesion depth with clinical cavitation have consistently shown that enamel lesions are frequently non-cavitated, whereas the likelihood of cavitation increases as radiolucency extends into dentin [9,10,11,12]. This association was demonstrated in a cross-sectional study of 1717 approximal surfaces in adults, in which cavitation was assessed visually following temporary tooth separation [38]. Cavitation was detected in only 0.2% of E1 (outer enamel) lesions and 6.1% of E2 (inner enamel) lesions, compared with 14.8% of D1, 50.0% of D2, and 61.5% of D3 lesions. Importantly, 65.4% of lesions radiographically limited to dentin (D1 to D3) showed no evidence of cavitation, indicating that radiographic depth alone is a poor predictor of cavitation, particularly for D1 and D2 lesions [39,40]. These findings support a management strategy that emphasizes preventive, non-invasive measures, including plaque control, remineralization, and monitoring, for enamel and D1 lesions. For D1 and D2 lesions, temporary tooth separation may provide valuable additional information to guide clinical decision making, whereas restorative intervention should generally be reserved for lesions with confirmed cavitation, especially those classified as D3 [10]. These data support a conservative threshold in which initial enamel lesions are typically managed with preventive or microinvasive approaches, while cavitated or plaque retentive lesions are more likely to require restorative intervention.

### 4.2. Caries Risk Assessment and the Threshold for Intervention

Caries risk assessment constitutes an essential component of clinical decision-making, as lesions presenting similar radiographic features may exhibit divergent biological behavior depending on the patient’s disease activity, protective factors, dietary habits, fluoride exposure, salivary conditions, oral hygiene practices, and previous caries experience [41,42,43,44]. Its clinical value lies in supporting individualized caries management, facilitating early lesion detection, strengthening evidence-based treatment decisions, and ultimately contributing to improved long-term oral health outcomes [4].

It should be emphasized, however, that caries risk assessment does not replace individual lesion assessment; instead, it shifts the intervention threshold along a continuum rather than acting as a fixed radiographic cut-off. In practice, inactive, non-cavitated lesions may be appropriately managed through periodic monitoring and preventive care, whereas high caries activity warrants closer surveillance and a lower threshold for micro-invasive intervention [45]. Despite this evidence-based rationale, a recent multinational survey of nearly 3700 dentists found that adherence to risk-based, conservative thresholds remains low and highly variable across countries, with most clinicians initiating invasive treatment well before the recommended threshold, particularly in low-risk patients [46].

In this context, based on the integration of RCT evidence and expert clinical consensus, resin infiltration assumes relevance for active, non-cavitated proximal lesions, especially when lesion accessibility is limited, progression is suspected, or the patient presents a moderate-to-high caries risk profile. Splieth et al. [45] reported that micro-invasive treatment effectively arrests non-cavitated enamel lesions and initial dentinal lesions limited to the outer third of dentin and is significantly more effective than non-operative professional care (e.g., fluoride varnish) or oral hygiene advice (e.g., flossing instruction) alone, particularly in higher-risk groups such as adolescents. This sequential approach positions resin infiltration as an intermediate strategy between preventive and restorative care within the broader spectrum of minimally invasive dentistry [3,7,41].

**Figure 1:**
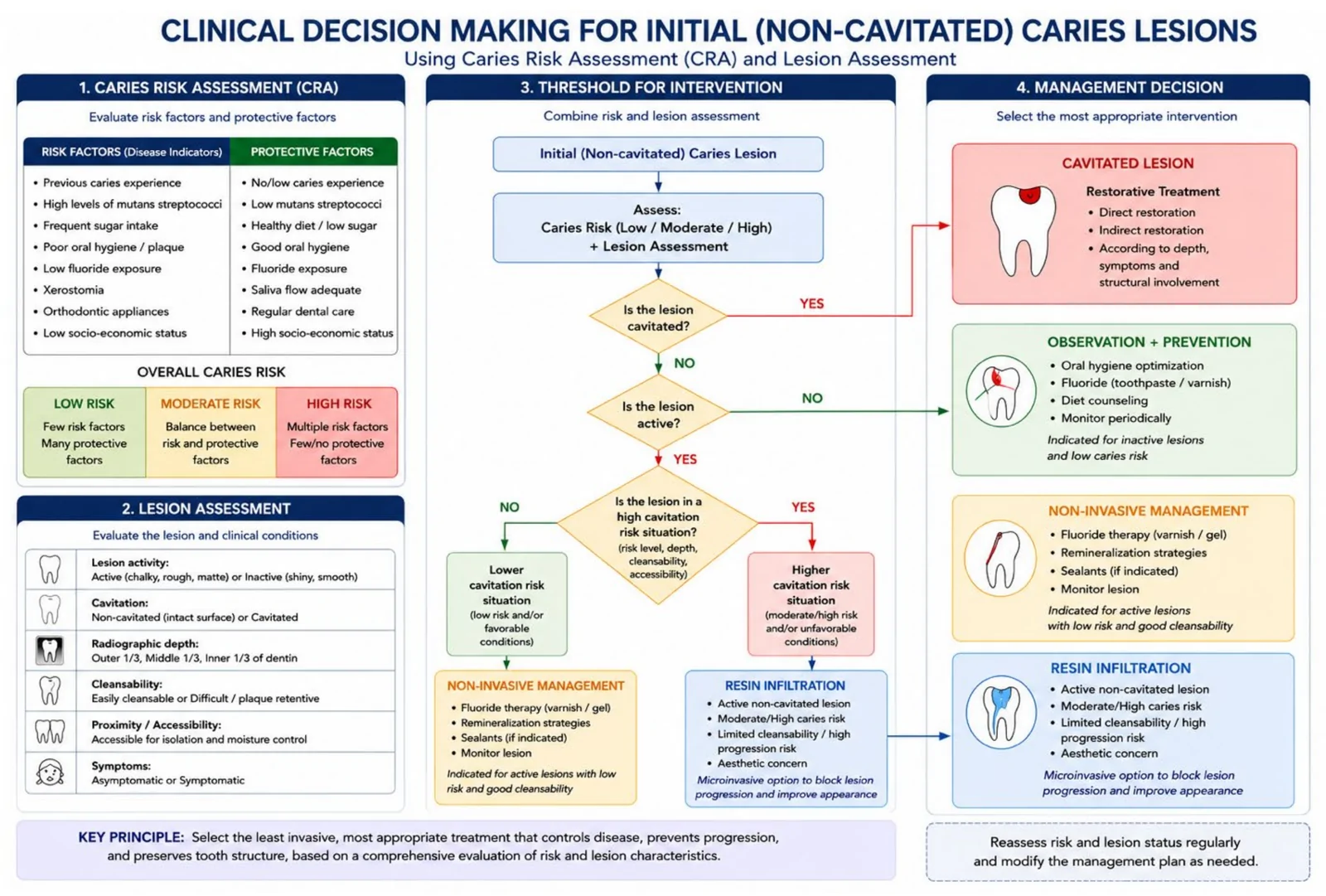
Proposed clinical pathway for the management of initial proximal caries lesions, incorporating patient-level caries risk, lesion activity status, and radiographic depth as decision criteria for non-invasive, microinvasive, or operative intervention.

The decision to infiltrate should therefore follow a two-stage logic. First, the lesion must be evaluated for activity, cavitation, cleanability, and depth. Second, the patient risk profile should be used to estimate the likelihood of new lesion development or progression. Resin infiltration is most rational when the lesion is non-cavitated, active or progressing, difficult to clean or monitor, and present in a patient whose risk profile suggests that prevention alone may be insufficient. This two-stage decision process, integrating lesion assessment with caries risk assessment, is summarized in Figure 1.

Table 1 summarizes how lesion assessment and caries risk assessment combine to guide management decisions, including the specific situations in which resin infiltration is the preferred option.

## 5. Principles and Clinical Protocol of Resin Infiltration

### 5.1. Mechanism of Action

Resin infiltration is based on the penetration of a low-viscosity, unfilled resin into the porous body of an enamel lesion following conditioning of the hypermineralized surface layer, typically achieved through etching with hydrochloric acid gel and subsequent dehydration with ethanol. The infiltrant is then drawn in by capillary action and polymerized in situ under a curing light. In vitro confocal laser scanning microscopy has demonstrated that the resin can reach up to 96% of lesion depth, confirming substantial sealing of the porous lesion body under laboratory conditions [47]. Once polymerized, the infiltrant blocks diffusion pathways for acids and dissolved minerals, thereby reducing permeability, supporting caries arrest, and reinforcing the weakened enamel structure. Translating this laboratory mechanism to the clinical setting, the diffusion-barrier effect underlies the reduction in radiographic lesion progression reported in clinical studies, with long-term meta-analyses showing 60–80% reductions in progression risk compared with observation or fluoride varnish [48,49].

Because treatment success depends on adequate resin uptake, lesion depth is an important determinant of outcome. Consistent with these laboratory findings on depth-dependent penetration, clinical evidence from a systematic review and meta-analysis of randomized controlled trials, Liang et al. [50] found resin infiltration highly effective for non-cavitated lesions confined to enamel and still beneficial at the enamel-dentin junction, but its effect was no longer statistically significant once lesions reached the outer third of dentin, indicating declining efficacy with increasing depth. This pattern is consistent with Liu et al. [51], who reported approximately 99.1% resin penetration in enamel compared with 82.0% in dentin. The reduced uptake in dentin likely reflects differences in the composition, histology, and caries pathology of the two tissues.

### 5.2. Clinical Protocol

Supported by the methodology reported across multiple randomized controlled trials, the clinical protocol most reported in the literature comprises the following sequential steps: case selection, plaque removal, isolation, acid conditioning, ethanol-mediated drying, application of the resin infiltrant, light-curing, reapplication when clinically indicated, and final finishing and polishing [21,45,47]. Most studies employed 15% hydrochloric acid for surface conditioning, followed by ethanol drying and application of a commercially available infiltrant system (e.g., ICON^®^). For proximal lesions specifically, temporary elastic separation is typically placed, with the patient recalled after approximately 24 h once adequate interproximal separation has been achieved, thereby allowing direct access for surface conditioning and infiltrant application. Variations in protocol, including differing conditioning regimens, application times, and adjunctive pretreatments, have nonetheless been reported across the literature, reflecting the methodological heterogeneity noted in systematic reviews of proximal lesion management [45].

**Figure 2:**
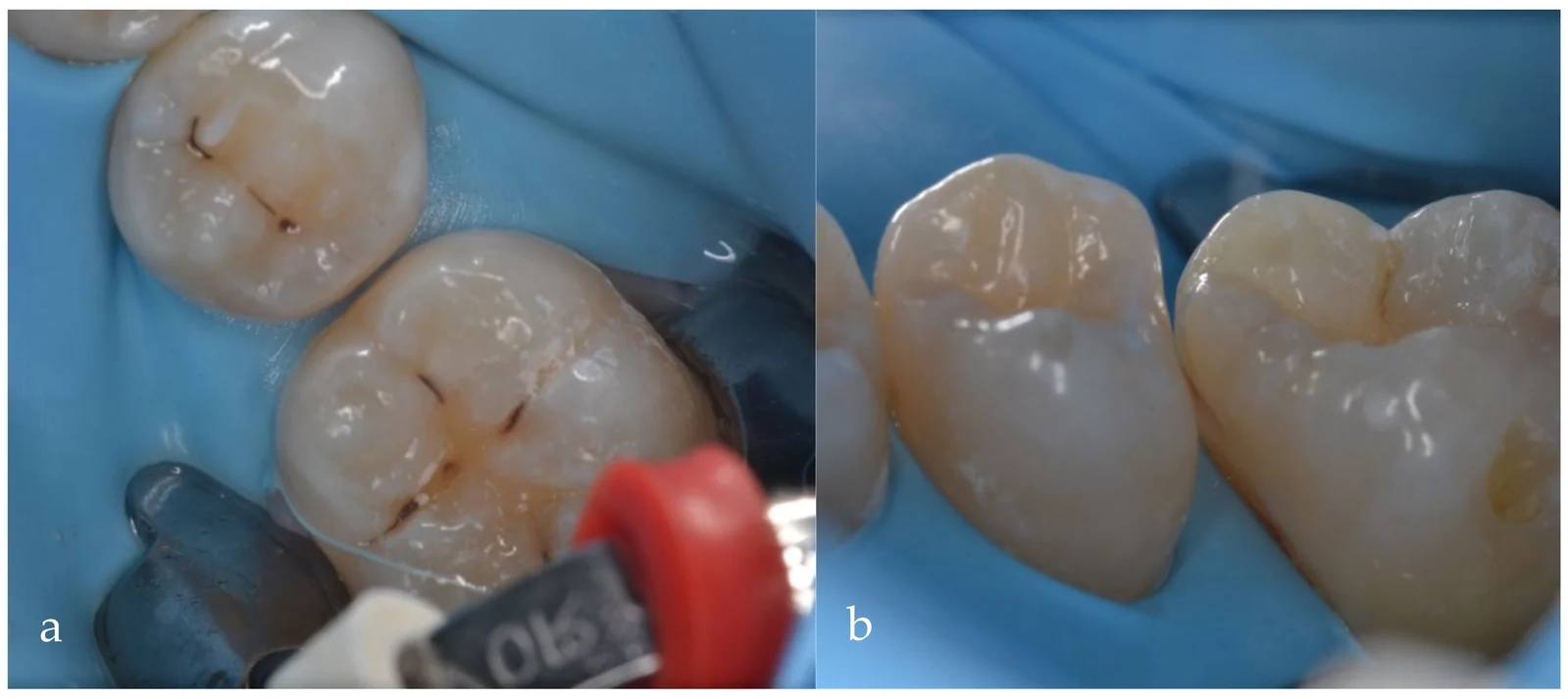
Occlusal and lateral views of the teeth following 24 h of temporary elastic separation (**a**), demonstrating the opened proximal contact at the recall visit and the incipient proximal lesion on the mesial surface of the maxillary first molar (**b**).

**Figure 3:**
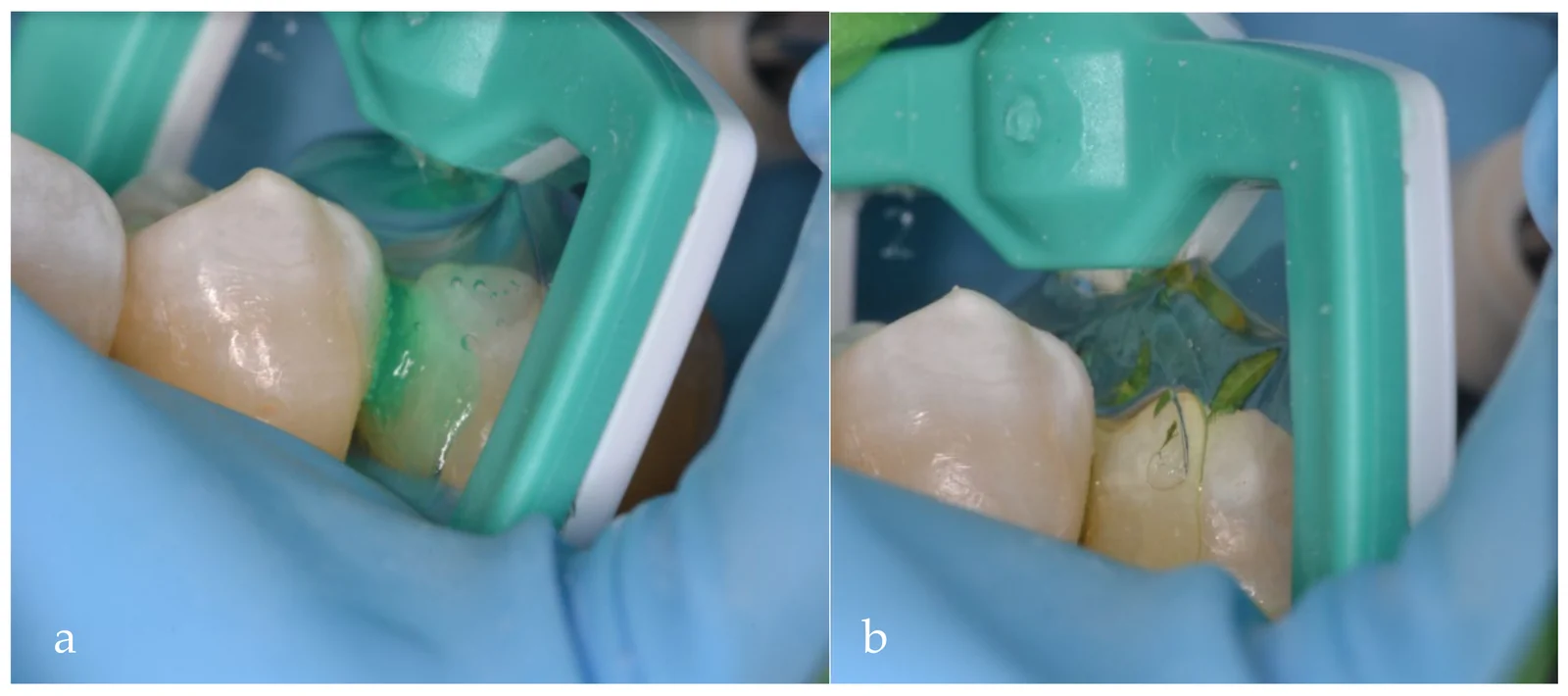
View of the 15% hydrochloric acid (HCl) gel applied to the lesion area (**a**), followed by application of the low viscosity resin infiltrant (**b**) after completion of the preceding water rinsing and 99% ethanol (Icon Dry) drying steps.

To complement the discussion of the clinical technique, representative images obtained during an ethically approved clinical research project illustrate selected procedural steps of resin infiltration for a proximal lesion (Figure 2a,b and Figure 3a,b).

### 5.3. Technique Sensitivity Factors

Technique sensitivity remains a clinically relevant consideration. Factors such as moisture control, adequacy of surface conditioning, sufficiency of drying, infiltration duration, and polymerization may all influence resin penetration and treatment outcome. The mode and frequency of acid-conditioning applications are similarly important, as repeated or active hydrochloric acid application may result in additional enamel removal and should therefore be weighed against the principles of minimal intervention [52,53]. Lesion characteristics constitute a further determinant of treatment success. Laboratory evidence suggests that acute white-spot lesions infiltrate more predictably than chronic, pigmented lesions, the latter potentially containing organic material, mineral deposits, or surface barriers that impede complete resin penetration [21,52,54,55,56,57,58,59,60]. Collectively, these factors underscore the importance of rigorous case selection and realistic communication with patients regarding anticipated treatment outcomes [61].

## 6. Synthesis of Clinical Evidence

This review positions resin infiltration as a selective micro-invasive treatment for incipient, non-cavitated proximal lesions in permanent teeth. Supported by RCT evidence and clinical consensus, resin infiltration is most clearly indicated when a proximal lesion is non-cavitated, active or progressing, and difficult to manage predictably with prevention alone. The decision is not purely radiographic; it requires integration of lesion activity, cavitation status, cleansability, radiographic depth, patient risk, and patient preference. Caries risk assessment is therefore central, as it modifies the threshold at which clinicians move from observation or prevention toward micro-invasive intervention [42,43].

Updated systematic reviews of nonrestorative caries treatment continue to report high arrest rates for resin infiltration in non-cavitated proximal lesions, with the strongest evidence found in permanent teeth when permanent-dentition data can be separated from primary-tooth findings [49,50,62,63,64,65,66,67,68]. The Cochrane review by Dorri et al. concluded that micro-invasive treatment of proximal lesions reduces the odds of progression relative to non-invasive management [62], while subsequent meta-analyses by Liang et al. and Chatzimarkou et al. quantified this benefit and examined its dependence on lesion depth and follow-up duration [50,64]. A trial sequential analysis by Cebula et al. reported that the evidence for caries arrest in both permanent and primary teeth had reached a level of firmness uncommon in operative dentistry, strengthening confidence in the proximal indication [49], and updated nonrestorative treatment reviews continue to rank resin infiltration among the more effective options for approximal lesions [68,69]. Because several of these reviews pooled primary and permanent dentition, this manuscript interprets those findings cautiously, emphasizing that permanent-tooth estimates, although consistent, rest on a smaller number of trials than the headline pooled figures suggest; Table 2 summarizes the key systematic reviews and meta-analyses informing this synthesis.

The most clinically relevant evidence for caries arrest concerns non-cavitated proximal lesions in permanent teeth, where long-term and pragmatic clinical studies consistently report reduced radiographic progression following resin infiltration compared with control or non-invasive approaches [11,12,14,70,71,72,73,74,75,76]. The most robust single study remains the seven-year randomized split-mouth trial by Paris et al. [11], in which infiltrated proximal lesions showed substantially less radiographic progression than placebo-treated controls across ICDAS codes (9% vs. 45%), with a stable protective effect and no adverse events. Shorter-term trials corroborate this pattern in the same direction of effect: Peters et al. observed comparable progression rates after three years (14% vs. 48%), Arslan and Kaplan reported an 80–90% relative risk reduction after one year, and Todorova and Filipov found significantly lower progression in infiltrated lesions at both one year (3% vs. 28.3%) and two years (3% vs. 33.3%), with remineralization showing an intermediate, non-significant benefit relative to no treatment [12,70,71,72]. Practice-based and more recent clinical studies further confirm this relevance in routine care [73,74]. Taken together, these findings demonstrate a consistent and durable protective effect of resin infiltration against proximal lesion progression across follow-up periods, supporting resin infiltration as a treatment option for enamel and selected outer-dentin proximal lesions when the surface remains non-cavitated.

Two qualifications emerge when these findings are weighed against the broader literature. First, the magnitude of benefit is depth-dependent: lesions confined to enamel or reaching the enamel-dentin junction respond more predictably than deeper lesions, a pattern reported across depth-stratified analyses [11,50]. Second, the comparator matters: where infiltration has been compared directly with fluoride varnish, the infiltrant has generally shown superior progression control for established non-cavitated proximal lesions [14], although fluoride-based regimens remain appropriate for shallower or inactive lesions and for patients in whom broader disease control is the dominant clinical need. These nuances support a depth- and activity-based indication rather than a blanket recommendation; the intervention should be viewed as part of a broader disease-control strategy rather than a substitute for oral hygiene instruction, fluoride exposure, dietary counseling, and recall.

Table 3 summarizes representative clinical studies of caries arrest and lesion progression in permanent teeth, including study design, lesion type, comparator, and follow-up period.

Two trials in Table 3 merit explicit discussion as important qualifications to the predominantly positive evidence base. Arthur et al. [13] conducted a three-year split-mouth randomized controlled trial in which resin infiltration showed no statistically significant additional benefit over placebo when background caries activity was well controlled (7.4% vs 18.5% progression, *p* = 0.453). This finding does not contradict the overall evidence for resin infiltration but reinforces the critical importance of patient selection: the technique is most appropriate when disease activity is not adequately controlled by prevention alone, and its additional benefit may be limited in patients who are already well managed preventively. Kaya et al. [77] reported that in moderate-to-high caries risk adults receiving motivational instruction alongside treatment, resin infiltration and fluoride varnish achieved equivalent arrest rates of 98%, with no statistically significant difference between groups. This finding supports the view that resin infiltration is a selective rather than universal intervention, and that the adjunctive care context, particularly the intensity of preventive counseling provided alongside treatment, materially influences the relative clinical benefit. Taken together, these trials reinforce the individualized, risk-based approach advocated throughout this review.

### Laboratorial Evidence

Systematic and laboratory studies demonstrate that low-viscosity infiltrants penetrate demineralized enamel, reduce its permeability, and increase surface hardness while reducing surface roughness, supporting the diffusion-barrier concept that underlies caries arrest [52,54,55,59]. Confocal microscopy studies confirm substantial mean penetration of commercial infiltrant into demineralized enamel [59], and reviews of penetration depth emphasize that the completeness of penetration, rather than its mere occurrence, governs the durability of the barrier [55]. Penetration is not uniform across all lesions, however: factors such as lesion age, mineral distribution, organic content, degree of surface obstruction, pretreatment protocol, and drying technique may all influence resin uptake [55,60,81,82,83,84,85], and penetration and sealing are less complete in older or more mineralized lesions [60]. This is clinically relevant for proximal lesions, because incomplete penetration leaves residual porosity through which demineralization can continue. While this rationale originates from laboratory evidence, it is consistent with the pattern of clinical benefit observed in trials, which have shown the clearest effect in active, recently formed lesions.

At the same time, in vitro data temper expectations about the extent to which resin infiltration restores the mechanical properties of demineralized enamel. Acid-challenge testing indicates that resin infiltration reduces surface roughness but does not restore the microhardness lost to demineralization, with the commercial infiltrant maintaining more stable properties than experimental formulations following acid challenge [20]. In other words, in vitro evidence indicates that infiltration stabilizes and seals the lesion rather than rebuilding mineral; this is consistent with, and helps explain, its role as a progression-control measure rather than a restorative one. Table 4 summarizes the mechanistic and laboratory evidence, including specimen type, penetration depth, and surface hardness or roughness outcomes informing clinical interpretation.

## 7. Limitations

Although this technique has shown promising results, clinicians should be aware of several practical limitations. First, the protocol for proximal incipient lesions requires a two-visit sequence in which temporary elastic separators are placed and the patient returns after approximately 24 h for surface conditioning and infiltration [11,37]. Patient cooperation is therefore a prerequisite for successful treatment; reluctance to return for the second visit may limit the feasibility of the proximal technique in less adherent populations.

Second, no discernible difference can be observed between pre- and post-treatment radiographs. This finding is consistent with the non-radiopaque nature of the currently available commercial infiltrant formulation: the resin is essentially an unfilled, low-viscosity material based on triethylene glycol dimethacrylate (TEGDMA) and does not contain radiopaque inorganic fillers, rendering it radiolucent and incapable of altering the radiographic appearance of the lesion. Moreover, the intended radiographic endpoint of proximal infiltration is the absence of lesion progression over time rather than any immediate radiographic change; a stable radiograph therefore represents the expected outcome rather than direct evidence of treatment efficacy.

Third, no visible difference is observed in the clinical appearance of the proximal lesion between pre- and post-treatment evaluation. Although the treatment goal for incipient proximal lesions is arrest of the lesion rather than esthetic masking, the absence of any clinical or radiographic change means there is no direct evidence confirming correct application of the infiltrant; the treatment leaves no detectable signature. A clinician subsequently examining the tooth or radiograph would therefore be unable to determine that resin infiltration had previously been performed. This finding carries important implications for clinical record-keeping and continuity of care, underscoring the necessity of thoroughly documenting the intervention within the patient record.

Fourth, the available evidence includes important negative and neutral findings that qualify the predominantly positive picture. Arthur et al. [13] found no statistically significant benefit of resin infiltration over placebo in a three-year randomized trial when caries activity was well controlled, suggesting that the technique’s benefit is conditional on the patient’s background disease activity. Kaya et al. [77] found equivalent arrest rates between resin infiltration and fluoride varnish when motivational instruction was provided alongside both treatments. These findings highlight that resin infiltration should not be viewed as a replacement for comprehensive preventive care, and that its incremental benefit depends on the clinical context in which it is applied.

Fifth, patient cooperation and adherence are prerequisites for successful treatment. The two-visit proximal protocol requires patients to tolerate elastic separator placement and return for a second appointment approximately 24 h later. In patients with low treatment compliance, dental anxiety, or limited access to care, this protocol may not be feasible, and simpler single-visit alternatives should be considered.

Sixth, the cost-effectiveness of resin infiltration relative to other non-invasive strategies across different risk profiles has not been extensively studied. Economic modelling by Schwendicke et al. [80] demonstrated that resin infiltration is cost-effective for high caries risk patients by reducing the frequency and complexity of future restorative interventions over a lifetime horizon. However, formal comparative cost-effectiveness analyses against fluoride varnish and other non-invasive approaches in low- and moderate-risk populations remain limited. Economic considerations should therefore be integrated into individualized clinical decision-making alongside clinical evidence.

Finally, the learning curve associated with resin infiltration has not been formally quantified in the literature. The two-visit proximal protocol involves multiple sequential steps, including elastic separation, surface conditioning, ethanol drying, infiltrant application, and light curing, each of which may influence the final outcome. While practice-based studies [73] support reproducibility across clinical settings and operator skill levels, the number of cases required to achieve consistent results has not been established, representing a recognized gap in the current evidence base.

## 8. Future Directions

Resin infiltration is evolving from a simple pore-sealing resin toward multifunctional infiltrants. It is important to note that the commercially available infiltrant (ICON, DMG, Hamburg, Germany) remains the only clinically validated system; all formulations discussed in this section are experimental and have not undergone clinical validation. They are presented as emerging research directions rather than established treatment alternatives. Beyond commercially available infiltrants, experimental formulations are being developed to extend the performance of resin infiltration. A literature review by Mazzitelli et al. cataloged a wide range of experimental modifications to the original TEGDMA-based formulation, including hydrophobic monomers (BisEMA, UDMA), alternative solvents, antibacterial agents, and bioactive fillers, noting that ethanol-containing formulations consistently showed impaired mechanical properties and penetration due to interference with polymerization, while TEGDMA/UDMA combinations and ethanol-free alternatives improved mechanical performance [21] A recent chemistry-focused review by Yang et al. further framed these efforts at the level of base monomer design, showing that structural modifications of Bis-GMA and UDMA (e.g., substitution of hydroxyl groups, incorporation of bulky or fluorinated side chains, urethane and silyl derivatives) can independently reduce polymerization shrinkage, lower viscosity, and decrease water sorption, while bio-based alternatives such as isosorbide-derived monomers aim to eliminate bisphenol A-related biosafety concerns; these monomer-level strategies represent a parallel avenue to filler-based bioactivity for improving infiltrant performance [86,87].

These include infiltrants modified with bioactive or remineralizing components such as hydroxyapatite, bioactive glass, and calcium phosphate compounds, as well as formulations designed to add radiopacity or antibacterial activity [21,86]. Pedreira et al. incorporated 40% ytterbium oxide into experimental and commercial (Icon) infiltrants, achieving radiopacity equal to or greater than enamel while improving polymerization and maintaining low solubility. Various approaches have been investigated, including quaternary ammonium monomers, ionic liquid systems, graphene oxide, and bioactive fillers. The incorporation of the pH responsive monomer DMAEM into a BisGMA/TEGDMA infiltrant significantly reduced bacterial activity and biofilm formation [30]. To enhance the resin infiltrants with bioactive properties, the addition of Biosilicate (5% or 10%) or nanohydroxyapatite (10%) induced calcium and phosphate deposition without impairing infiltration into white spot lesions [34]. Dai et al. incorporated amorphous calcium phosphate nanoparticles (NACP) into a commercial infiltrant and found that a 30% NACP modification sustained long-term calcium and phosphorus ion release and improved cross-sectional enamel hardness after pH cycling without compromising biocompatibility or degree of conversion [88]. More recently, Wang et al. developed a bioactive resin infiltrant incorporating a high-phosphorus sol–gel-derived bioactive glass (PSC) within a TEGDMA/HEMA-based matrix, demonstrating calcium and phosphorus ion release and hydroxyapatite formation on XRD analysis, while maintaining degree of conversion, microhardness, elastic modulus, water sorption, biocompatibility, and penetration depth comparable to the commercial control [35]. A recent systematic and critical review of bioactive inorganic materials in resin infiltrants reported favorable effects on enamel surface properties in vitro but emphasized methodological heterogeneity and the absence of clinical validation [86]. These materials are not yet available for routine clinical use and were therefore not included in the effectiveness synthesis, but they represent a plausible direction for improving lesion penetration, mechanical reinforcement, radiographic detectability, antibacterial capacity, and long-term remineralization potential.

## 9. Conclusions

Resin infiltration is a microinvasive treatment positioned between noninvasive prevention and restorative intervention. In permanent teeth, the strongest evidence supports its use for incipient, non-cavitated proximal lesions, where long-term randomized and observational long-term studies demonstrate reduced radiographic progression. Resin infiltration should be considered part of an individualized caries management strategy rather than a universal alternative to prevention or restoration, and clinicians should bear in mind its radiographically and visually silent nature in proximal applications, which demands careful documentation and patient cooperation with the two-visit protocol.

Material development is progressing along two fronts: optimization of base monomer chemistry to improve shrinkage, viscosity, and biosafety, and incorporation of bioactive, remineralizing, radiopaque, or antibacterial components to extend the infiltrant’s effect beyond pore sealing. These experimental formulations show favorable in vitro results but remain limited by methodological heterogeneity and the absence of clinical validation, so treatment decisions should continue to rest on established indications and the currently validated commercial material.

## Figures and Tables

**Table 1:** Clinical decision framework integrating lesion assessment and caries risk assessment.

Clinical Finding	Patient Risk Context	Preferred Management	Role of Resin Infiltration
Inactive non-cavitated lesion	Any risk level, provided disease control is feasible	Monitor, reinforce prevention, recall	Usually not indicated
Active enamel lesion	Low risk	Preventive therapy and follow up	Consider only if progression or esthetic concern persists
Active non-cavitated proximal lesion	Moderate risk	Preventive therapy plus microinvasive treatment if indicated	Consider resin infiltration
Active non-cavitated proximal lesion	High risk or difficult to clean	Risk factor control plus microinvasive stabilization	Resin infiltration often preferred if non-cavitated
Cavitated, plaque retentive lesion	Any risk level	Restorative treatment with disease control	Not a substitute for restoration

**Table 2:** Key systematic reviews and meta-analyses supporting the evidence synthesis.

Review	Scope	Population	Main Outcome	Key Message
Dorri et al. [62]	Microinvasive interventions, Cochrane	Primary and permanent	Lesion progression	Supports microinvasive care for proximal lesions
Domejean et al. [63]	RI for non-cavitated lesions	Permanent, in vivo	Caries arrest	Supports RI efficacy for non-cavitated lesions
Liang et al. [50]	Proximal caries by depth	Permanent focus	Progression by depth	RI effective for enamel and enamel dentin junction lesions
Cebula et al. [49]	RI proximal, trial sequential analysis	Primary and permanent	Progression	Firm evidence for caries arrest in permanent teeth
Urquhart et al. [68]	Nonrestorative treatments, network MA	Primary and permanent	Arrest or reversal	RI with fluoride varnish among most effective for approximal lesions
Cabalen et al. [69]	Nonrestorative caries treatment update	Primary and permanent	Arrest	High arresting rates for RI in non-cavitated proximal lesions

**Table 3:** Representative clinical studies on caries arrest and lesion progression in permanent teeth.

Study	Design	Lesion Type	Comparator	Follow Up	Main Outcome
Paris et al. [11]	RCT	Proximal E2 or D1	Placebo	7 years	Reduced progression, strong long-term evidence
Meyer-Lueckel et al. [70,71]	Pragmatic RCT	Proximal lesions	Noninvasive care or placebo	18 months to 4 years	Reduced progression, real world relevance
Peters et al. [12,72]	RCT	Proximal lesions	Placebo	2 to 3 years	Reduced radiographic progression
Arslan and Kaplan [14]	Clinical trial	Proximal lesions	Fluoride varnish	1 year	RI superior in progression control
Tiuraniemi et al. [73]	Practice based follow up	Interproximal lesions	NR *	Practice based	Supports proximal use in routine care
Todorova and Filipov [74]	Clinical study	Initial proximal lesions	Fluoride varnish	1 and 2 years	Lesion arrest or stabilization, recent evidence
Arthur et al. [13]	Split-mouth RCT	Proximal E1–D1, permanent teeth	Placebo (caries activity controlled)	3 years	No significant additional benefit of RI when caries activity is controlled; 7.4% vs 18.5% progression (*p* = 0.453)
Kaya et al. [77]	Parallel RCT	Non-cavitated proximal lesions, permanent teeth	Fluoride varnish	18 months	No significant difference between RI and fluoride varnish; both 98% arrest rate; motivational instructions beneficial
Abdalla et al. [78]	Prospective cohort	Non-cavitated and micro-cavitated lesions (ICDAS 1–4), adults	Observational (no RI)	2 years	24% of active non-cavitated and 31% of micro-cavitated lesions progressed; active and more severe lesions more likely to progress; proximal > smooth surface
Singh et al. [79]	Multi-arm RCT	Proximal enamel lesions, permanent teeth	Sealant; placebo	9 months	RI most effective: 70% stabilized vs 50% (sealant) vs 10% (placebo); lowest DMFT, DMFS and ICDAS scores throughout
Abdalla et al. [80]	Prospective cohort	Non-cavitated and micro-cavitated lesions (ICDAS 1–4), adults	Observational (no RI)	2 years	24% of active non-cavitated and 31% of micro-cavitated lesions progressed; active and more severe lesions more likely to progress; proximal > smooth surface
Singh et al. [81]	Multi-arm RCT	Proximal enamel lesions, permanent teeth	Sealant; placebo	9 months	RI most effective: 70% stabilized vs 50% (sealant) vs 10% (placebo); lowest DMFT, DMFS and ICDAS scores throughout

* NR = not reported.

**Table 4:** Laboratory evidence supporting clinical interpretation.

Study	Specimen or Method	Penetration	Hardness or Roughness	Key Message
Soveral et al. [54]	SR and meta-analysis	Pooled	Improved properties	RI improves enamel surface properties
Ibrahim et al. [55]	SR	Variable	Not assessed	Penetration depends on lesion and technique factors
Srikumar et al. [59]	CLSM, human enamel	High mean penetration	Not assessed	Strong penetration into demineralized enamel
Arora et al. [57]	Profilometry and hardness	Highest among groups	Favorable	ICON showed strong overall performance
Gevkaliuk et al. [60]	SEM, acute vs chronic	Higher in acute lesions	Not reported	Chronic pigmented lesions less predictable
De Cerqueira et al. [20]	Acid challenge, profilometry and hardness	Qualitative	Roughness reduced, hardness not restored	ICON more stable than experimental infiltrant

SR = systematic review; CLSM = confocal laser scanning microscopy; SEM = scanning electron microscopy.

## Data Availability

Additional data are available from the corresponding authors on reasonable request.
